# Specificities of the Use of Psychotropic Drugs in Bariatric Surgery

**DOI:** 10.1192/j.eurpsy.2022.1216

**Published:** 2022-09-01

**Authors:** C. Fernandes Santos, R. Gomes

**Affiliations:** Hospital Garcia de Orta, Department Of Psychiatry And Mental Health, Almada, Portugal

**Keywords:** psychopharmacology, obesity, bariatric surgery, psychotropics

## Abstract

**Introduction:**

Bariatric surgery is considered an effective treatment against obesity. Psychiatric illness is relatively common in patients who have undergone bariatric surgery. Over one-third of these patients are prescribed psychotropic drugs, particularly antidepressants. Unlike medications for diabetes, hypertension or hyperlipidemia, which are generally reduced and at times discontinued, postsurgery psychotropic use is only slightly reduced. The surgical intervention and the subsequent weight loss can affect several pharmacokinetic parameters, leading to a possible need of dosing adjustment.

**Objectives:**

To review the influence of bariatric surgery on the use and pharmacokinetics of psychotropic drugs.

**Methods:**

Non-systematic review of literature through search on *PubMed/MEDLINE* for publications from 2011 to 2021, following the terms *psychotropic* and *bariatric surgery.* Textbooks were consulted.

**Results:**

It is difficult to predict how psychotropics will be affected by bariatric surgery because of interindividual differences and limited data. Malabsorptive surgical procedures have a relatively greater potential to alter drug exposure. Medication disintegration, dissolution, absorption, metabolism and excretion have been found to be altered in postbariatric patients. Antidepressants are the best studied psychotropics in the bariatric population and their absorption is reduced. The risk of gastric bleeds with bariatric surgery will probably be increased by serotoninergic antidepressants. Antipsychotics and mood stabilisers are not well studied in these patients. Depot antipsychotics avoid the risk of reduced absorption after surgery. Lithium use requires particular close monitoring.

**Conclusions:**

Close treatment monitoring and the ongoing monitoring of symptoms are needed after bariatric surgery. Many patients may not require significant changes to drug treatment after surgery.

**Disclosure:**

No significant relationships.

